# Negative genic switch of HER-2 in the primary tumor instead of the synchronous metastatic nodal lesions after neoadjuvant chemotherapy in a patient with primary HER2-positive breast cancer

**DOI:** 10.1186/s12957-017-1255-8

**Published:** 2017-10-19

**Authors:** Hao-ran Chen, Yu-tuan Wu, Qiu-bo Yu, Ya-ying Yang, Yu-xian Wei, Hong-yuan Li, Kai-nan Wu, Ling-quan Kong

**Affiliations:** 1 Department of Endocrine & Breast Surgery, The First Affiliated Hospital of Chongqing Medical University, No.1, Youyi Road, Yuanjiagang, Yuzhong District, Chongqing, 400016 China; 2 Center for Molecular Medicine Testing, Chongqing Medical University, No.1, Yixueyuan Road, Yuanjiagang, Yuzhong District, Chongqing, 400016 China; 3 Clinical Diagnostic Pathology Center, Chongqing Medical University, No.1, Yixueyuan Road, Yuanjiagang, Yuzhong District, Chongqing, 400016 China

**Keywords:** Breast cancer, Axillary lymph node metastases, Neoadjuvant chemotherapy, Genic switch of HER2

## Abstract

**Background:**

A few retrospective studies have indicated that neoadjuvant chemotherapy (NAC) in breast cancer may change biomarker profiles of the primary tumor. Little is known about the status of HER-2 gene of the synchronous nodal metastases when that of the residual tumor undergoes negative conversion in a neoadjuvant setting.

**Case presentation:**

We describe a female patient with left breast cancer (T2N2M0) who underwent negative conversion of HER-2 in the primary tumor instead of the synchronous nodal lesions after NAC. Core needle biopsy showed invasive ductal carcinoma with HER2 immunohistochemistry (IHC) (2+) and amplified HER-2 gene determined by fluorescence in situ hybridization (FISH). Then, the patient underwent 4 cycles of anthracycline- and taxane-based NAC and subsequent left modified radical mastectomy. Postoperative pathology showed invasive ductal carcinoma involving 4 of 12 surgically excised axillary lymph nodes with HER2 IHC (1+) and FISH negative (HER2 gene not amplified) in the residual tumor of the breast specimen. Due to the negative genic switch of HER2 after NAC, the patient rejected to accept trastuzumab. Under the patient’s consent, the synchronous nodal lesions were further investigated and showed HER2 IHC(−) but FISH positive (HER-2 gene amplified). Therefore, the patient agreed to accept adjuvant trastuzumab treatment every 3 weeks for 1 year.

**Conclusions:**

We propose further assessment of HER2 gene in the synchronous nodal metastases, especially when negative genic switch of HER-2 occurs in the primary tumor after NAC in order to tailor the systemic regimens for breast cancer patients.

## Background

Neoadjuvant chemotherapy (NAC) is utilized in the context of locally advanced breast cancer to downstage tumors, improve operability, and increase the chance of breast-conserving surgery [1]. Patients receiving NAC shared equivalent disease progression and overall survival with those only receiving post-operative chemotherapy [2]. In early breast cancer, anthracycline and/or taxane-based regimens in a neoadjuvant setting provides information about the tumor’s sensitivity to chemotherapy and clinical outcomes after post-operative systemic therapy [3–5]. Approximately 15% of patients have achieved complete remission of the primary tumor and acquired better clinical outcomes by NAC [6, 7].

Recently, a trend has emerged in distinguishing prognostic factors by studying alterations of biomarkers in residual tumoral lesions. A few retrospective studies [8–11] have suggested that NAC in breast cancer may change biomarker profiles of the primary tumor. But, little is known about the status of the HER-2 gene of the synchronous nodal metastases after NAC when that of the residual tumor undergoes negative conversion [12], which poses a challenge to the inclusion of trastuzumab in the systemic therapy regimens. This issue has been significant in recent years due to the common usage of trastuzumab in HER-2 positive tumor, and decreased HER2 expression in invasive breast cancer after NAC has been noted [11, 13]. Given that sentinel lymph node biopsy or axillary lymph node dissection are performed in standard surgical practice and routine pathological assessment of nodes is carried out to evaluate the axillary surgical staging, this case report shows that there may be additional benefit to perform molecular testing on nodal metastases, especially when both HER-2 gene and its oncogenic receptor of the primary tumor underwent negative conversion after NAC.

Here, this case study reported on the loss of HER-2 gene in the primary focus of breast cancer after NAC and meanwhile, we found by FISH that HER-2 gene was still amplified in the synchronous metastatic axillary lymph nodes. Then, the patient agreed to accept trastuzumab every 3 weeks for 1 year.

## Case presentation

**Fig. 1:**
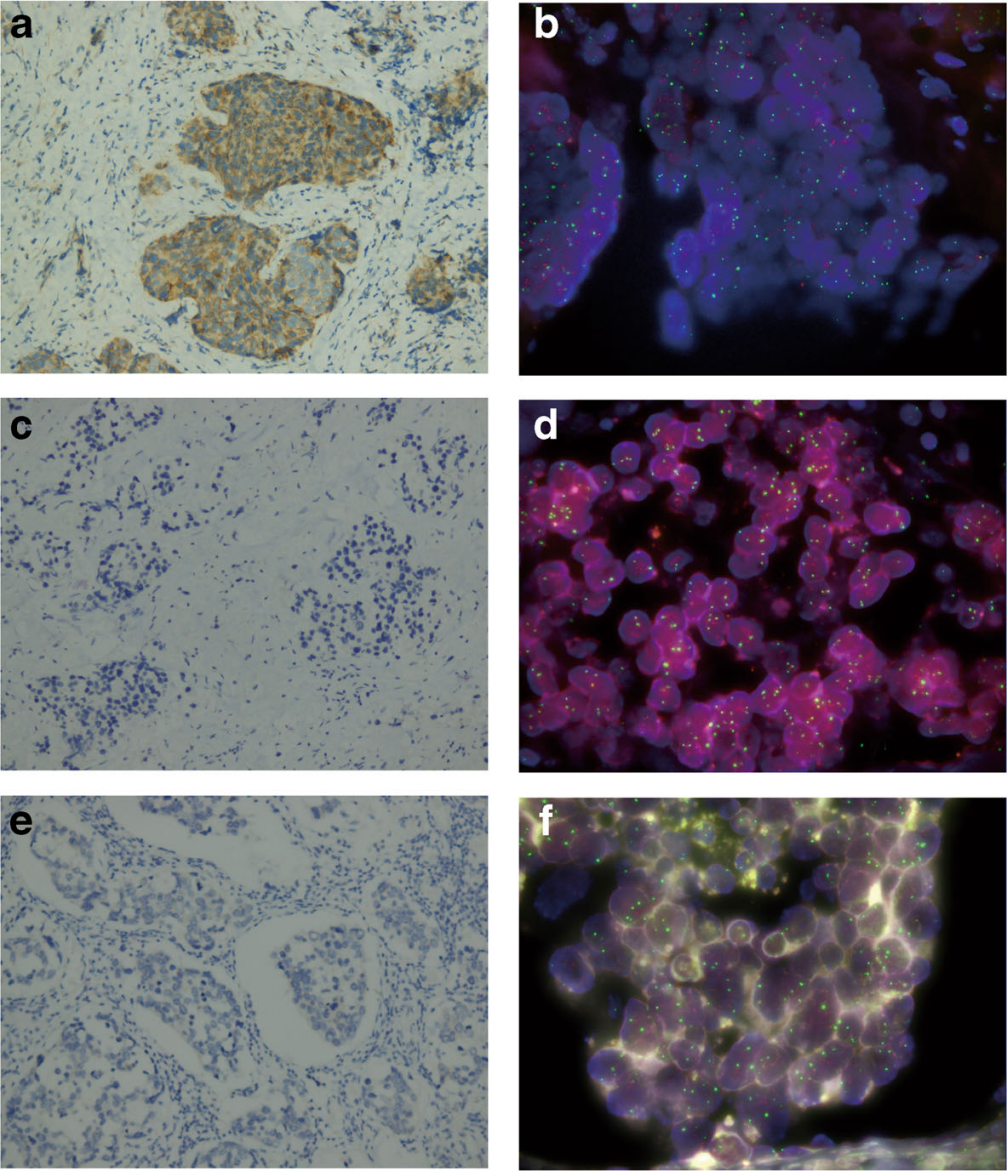
**a** HER-2 IHC (2+) of the core needle biopsied tissues of the primary tumor (IHC, *200). **b** Amplification of HER-2 gene (*HER-2 gene/CEP17 ratio: 2.34*) detected by FISH in core needle biopsied tissues of the primary tumor (green spots represent signals of centromeric chromosome 17 while red ones stand for that of HER-2 gene). **c** HER-2 IHC (−) of the surgically excised primary tumor after neoadjuvant chemotherapy (IHC, *200). **d** HER-2 gene of the surgically excised primary tumor after neoadjuvant chemotherapy was not amplified (*HER-2 gene/CEP17 ratio: 1.88, the average number of HER-2 gene per cell: 4.73*) as determined by FISH. **e** HER-2 IHC (−) of the synchronous metastatic axillary lymph nodes after neoadjuvant chemotherapy (IHC, *200). **f** HER-2 gene of the synchronous metastatic axillary lymph nodes was amplified (*HER-2 gene/CEP17 ratio: 2.21*) as determined by FISH. Intratumoral heterogeneity was indeed evaluated by observing the distribution of HER-2 gene within tumoral histological sections by fluorescent microscopy. The two signals were scattered in distribution without focal concentrations and clusters, showing no obvious spatial heterogeneity of HER-2 gene. One hundred cells of the tumoral histological sections were analyzed for HER-2 gene positivity. The total number of HER-2 gene signal and the average number of HER-2 gene that each cell harbored were counted and so were for CEP-17. HER-2 gene/CEP-17 ratio was calculated. According to the updated 2013 CAP/ASCO guideline on HER-2 testing [27], HER2/CEP17 ratio cutoff for amplification of HER-2 gene was lowered to ≥ 2.0 and an average HER-2 gene copy number criterion for amplification of HER-2 gene (≥ 6.0/cell) was introduced. *FISH testing protocol were obtained from Beijing GP Medical Technologies, Ltd./China Medical Technologies Inc. Beijing, China*

A 61-year-old female was hospitalized with a 1-month history of left-sided breast lump. Physical examination revealed a lump, 2 cm away from the nipple, about 4.0 × 3.0 cm in size, irregular on surface, hard in consistency, almost immobile and no tenderness in the outer upper quadrant of the left breast accompanied by a hard, fixed, and painless lymph node, about 2.0 cm × 1.5 cm in size, in the left axilla. Both breast ultrasonography and mammography supported the diagnosis of breast cancer. Then, she was diagnosed with breast cancer (T_2_N_2_M_0_) by core needle biopsy; no distant metastases were found during therapy and follow-up. Core needle biopsy of the mass revealed moderately differentiated invasive ductal adenocarcinoma of non-specific type. Immunohistochemistry (IHC) showed estrogen receptor (ER) 80% (+), progesterone receptor (PR) 40% (+), human epidermal growth factor receptor 2 (HER-2) (2+) (Fig. 1a), Ki67 20% (+), and P53 (−). HER-2 gene was amplified (*HER-2 gene/CEP17 ratio: 2.34*) as determined by fluorescence in situ hybridization (FISH) (Fig. 1b). Then, the patient underwent 4 cycles of NAC (*cycled every 21 days*) with TEC regimens (*epirubicin 75 mg/m* ^*2*^ *IV day1, cyclophosphamide 500 mg/m* ^*2*^ *IV day1, docetaxel 75 mg/m* ^*2*^ *IV day*2) followed by left modified radical mastectomy. Excised biopsy revealed moderately differentiated invasive ductal adenocarcinoma of non-specific type, grade 2, score of 6 points (*NOTTINGHAM COMBINED HISTOLOGICAL GRADE*) with left axillary lymph nodes metastases (4/12). IHC performed on the surgically excised primary tumor showed ER 60% (+), PR 5% (+), HER-2 (−) (Fig. 1c), ki67 5% (+), and P53 (−). HER-2 gene was shown to be non-amplified (*HER-2 gene/CEP17 ratio: 1.88*) as detected by FISH (Fig. 1d). Due to the negative conversion of HER-2 status of the primary tumor after NAC, the patient rejected to accept targeted therapy with trastuzumab. Under the patient’s consent, we further submitted the metastatic axillary lymph nodes for IHC, showing ER 90% (+), PR 60% (+), HER-2 (−) (Fig. 1e), ki67 20% (+), and P53 10% (+). FISH was finally performed on the synchronous metastatic axillary lymph nodes to detect the actual status of HER-2 gene, revealing that the HER-2 gene was still amplified (*HER-2 gene/CEP17 ratio: 2.21*) (Fig. 1f). The patient received trastuzumab treatment (8 mg/kg IV day1 followed by 6 mg/kg) every 3 weeks to complete a 1-year targeted therapy. She received local/regional irradiation and adjuvant hormonal therapy following adjuvant chemotherapy. There exists no evidence of recurrence in her follow-up clinical evaluation thus far, 1 year after diagnosis. All the pathology, IHC and FISH, were determined by the Clinical Diagnostic Pathology Center and Center for Molecular Medicine Testing of Chongqing Medical University of China.

## Discussion

In this patient, HER2 status of the primary tumor converted from positivity to negativity after NAC, which is in accordance with the results of one preclinical study [13] showing that paclitaxel downregulated HER2 expression in MDA-MB 453 cells and one retrospective study by Niikura et al. [14], which showed that 21.4% of HER-2 positive primary tumor had lost HER-2 expression in a neoadjuvant setting, and strongly supported the necessity to retest HER-2 status of residual tumor after neoadjuvant therapy in order to accurately determine appropriate utilization of anti-HER-2 therapy. The primary tumor of the patient after NAC was proved to be HER-2 negative by IHC and FISH. And then, the patient rejected to accept trastuzumab. Similarly, in the study of Niikura et al. [14], 28% of patients whose cancer lost HER-2 expression in a neoadjuvant setting did not receive trastuzumab treatment. Indeed, HER-2 status has been widely and successfully established in certified diagnostic pathology laboratories; potential diagnostic pitfalls of this “simple” marker can often occur in a neoadjuvant setting due to the modulatory effect of NAC on HER-2 expression of the primary tumor [8–12] and intratumoral heterogeneity [10, 15]. We appreciate the productive work of Niikura et al. [14] in throwing light upon the phenomenon of negative conversion of HER2 status in residual tumors after NAC and its potential influence on the inclusion of trastuzumab in systemic therapy regimens. In the absence of synchronous nodal metastases, their study bears much significance because negative genic switch of HER2 in residual tumors after NAC poses a challenge to the utilization of targeted therapy with a proportion of patients whose cancer lost HER2 expression after NAC in their study not receiving trastuzumab. But in the presence of synchronous nodal metastases, the situation becomes more complicated because metastatic tumor cells that originate from the HER-2 positive primary tumor prior to NAC may still harbor amplified HER2 gene. Various studies [16–18] have revealed certain discrepancies of HER2 expression between primary tumors and synchronous nodal metastases in the absence of chemotherapy, ranging from 0 to 9%. Little is known about the status of HER-2 gene of synchronous nodal metastases in a neoadjuvant setting, as confirmation of this oncogene in nodal lesions is crucial under the condition in which HER-2 gene status of the primary tumor converted from amplification to non-amplification after NAC [12]. So, we shifted our attention to the pathologically confirmed metastatic lesions within axillary lymph nodes (4/12). In order to optimize the patient’s clinical outcomes, the synchronous nodal metastases were submitted for IHC, only to find the nodal lesions shared the same negative HER-2 status with the primary tumor, which indicated that HER-2 protein expression of nodal lesions was also suppressed by NAC, as Oldham et al. [13] demonstrated that paclitaxel downregulated HER2 expression in MDA-MB 453 cells. Finally, FISH was performed and confirmed amplification of HER-2 gene of the synchronous nodal metastases. Then, the patient agreed to receive trastuzumab. This discrepancy between HER-2 gene and its oncogenic receptor as revealed by IHC and FISH, respectively, was in accordance with one study [19] indicating that the HER-2 expression at the transcriptomic level is not always parallel to that at the proteomic level based on ERBB2 mRNA analysis after neoadjuvant systemic therapy, thus emphasizing that conclusions drawn from proteomic level should be further investigated in genomic and transcriptomic level. We speculated that suppression of HER2 protein expression of the nodal lesions in a neoadjuvant setting in this patient was possibly contemporary and the oncogenic receptor would soon be regained following a cessation of chemotherapy, due to the persistent amplification of HER-2 gene in nodal lesions.

Aitken et al. [20] confirmed by breast tissue microarray (TMA) and matched node TMA that biomarkers’ status of metastatic nodal lesions could be a more accurate measurement for guiding adjuvant therapy. It is postulated that biomarker profiles expressed by locoregional nodal lesions are more indicative of the biological behaviors of distant micro-metastases and circulating tumor cells (CTCs) that are more invasive and closely correlated with the potential clinical outcomes [21, 22] and vice versa. Onsten et al. [23] elaborated two cases in which gene expression profiles of CTCs bore better resemblance to that of the nodal metastases than the primary tumor when molecular discordance between CTCs and the primary tumor were investigated by reverse transcription quantitative PCR. Aktas et al. [24] demonstrated that detection and evaluation of HER-2 status in CTCs by liquid biopsy was able to make relatively accurate prediction about HER-2 gene and its oncogenic receptor status on metastases in a prospective open non-randomized study. Investigation of the biology of CTCs is significant, as a classical genetic model of human cancer progression is provided by the analysis of CTCs development [25]. White et al. [26] demonstrated that disseminated tumor cells isolated at the time of diagnosis shared the same subclonal cell DNA copy number aberration with relatively large proportions of breast cancer cells in the lymph node metastases.

While the necessity to retest the HER2 status of residual tumors after NAC to tailor the utilization of anti-HER2 therapy is being emphasized [14], the possibility always lost sight that synchronous nodal metastases, distant micrometastases, and even CTCs, if any, which originate from the HER2 positive primary tumor, may still harbor tumor cells that are HER2-gene amplified.

## Conclusions

This case report highlights, in a neoadjuvant setting, the significance of further assessment of HER-2 gene in the synchronous nodal metastases, especially when negative genic switch of HER-2 occurs in the primary tumor in order to formulate a more tailored adjuvant therapy, thus improving the patient’s outcome. Even if HER-2 gene status of synchronous nodal metastases undergoes negative conversion, whether to adopt the protocol of trastuzumab-containing adjuvant therapy still remains uncertain, because we are still not sure whether it can completely represent the characteristics of the distant metastatic cancer cells or CTCs. Prospective studies of HER-2 gene analyses among various matched lesions (i.e., primary tumors, synchronous nodal metastases, distant micro-metastases, and CTCs) in a neoadjuvant setting are needed for further study. Furthermore, a randomized controlled trial on whether to adopt the protocol of trastuzumab-containing adjuvant therapy among patients whose cancer lose HER2 expression not only in the primary tumor but also in the synchronous metastatic nodal lesions after NAC should be carried out.

## References

[1] Kaufmann M, von Minckwitz G, Smith R (2003). International expert panel on the use of primary (preoperative) systemic treatment of operable breast cancer: review and recommendations. J Clin Oncol.

[2] Mauri D, Pavlidis N, Ioannidis JP (2005). Neoadjuvant versus adjuvant systemic treatment in breast cancer: a meta-analysis. J Natl Cancer Inst.

[3] Fisher B, Brown A, Mamounas E (1997). Effect of preoperative chemotherapy on local-regional disease in women with operable breast cancer: findings from National Surgical Adjuvant Breast and Bowel Project B-18. J Clin Oncol.

[4] Bear HD, Anderson S, Smith RE (2006). Sequential preoperative or postoperative docetaxel added to preoperative doxorubicin plus cyclophosphamide for operable breast cancer: National Surgical Adjuvant Breast and Bowel Project Protocol B-27. J Clin Oncol.

[5] Wolff AC, Berry D, Carey LA (2008). Research issues affecting preoperative systemic therapy for operable breast cancer. J Clin Oncol.

[6] Pierga JY, Mouret E, Laurence V (2003). Prognostic factors for survival after neoadjuvant chemotherapy in operable breast cancer. The role of clinical response. Eur J Cancer.

[7] Wolmark N, Wang J, Mamounas E et al. Preoperative chemotherapy in patients with operable breast cancer: nine-year results from National Surgical Adjuvant Breast and Bowel Project B-18. J Natl Cancer Inst Monogr. 2001;(30):96–102. 10.1093/oxfordjournals.jncimonographs.a003469 11773300

[8] Lorgis V, Algros MP, Villanueva C (2011). Discordance in early breast cancer for tumour grade, estrogen receptor, progesteron receptors and human epidermal receptor-2 status between core needle biopsy and surgical excisional primary tumour. Breast.

[9] Kumaki N, Umemura S, Tang X (2011). Alteration of immunohistochemical biomarkers between pre- and post-chemotherapy: hormone receptors, HER2 and Ki-67. Breast Cancer.

[10] Zhou X, Zhang J, Yun H (2015). Alterations of biomarker profiles after neoadjuvant chemotherapy in breast cancer: tumor heterogeneity should be taken into consideration. Oncotarget.

[11] Li P, Liu T, Wang Y (2013). Influence of neoadjuvant chemotherapy on HER2/neu status in invasive breast cancer. Clin Breast Cancer.

[12] Cockburn A, Yan J, Rahardja D (2014). Modulatory effect of neoadjuvant chemotherapy on biomarkers expression; assessment by digital image analysis and relationship to residual cancer burden in patients with invasive breast cancer. Hum Pathol.

[13] Oldham EA, Li C, Ke S (2000). Comparison of action of paclitaxel and poly(L-glutamic acid)-paclitaxel conjugate in human breast cancer cells. Int J Oncol.

[14] Niikura N, Tomotaki A, Miyata H (2016). Changes in tumor expression of HER2 and hormone receptors status after neoadjuvant chemotherapy in 21,755 patients from the Japanese breast cancer registry. Ann Oncol.

[15] Hanna WM, Ruschoff J, Bilous M (2014). HER2 in situ hybridization in breast cancer: clinical implications of polysomy 17 and genetic heterogeneity. Mod Pathol.

[16] Ataseven B, Gologan D, Gunesch A (2012). HER2/neu, topoisomerase 2a, estrogen and progesterone receptors: discordance between primary breast cancer and metastatic axillary lymph node in expression and amplification characteristics. Breast Care (Basel).

[17] Jensen JD, Knoop A, Ewertz M, Laenkholm AV (2012). ER, HER2, and TOP2A expression in primary tumor, synchronous axillary nodes, and asynchronous metastases in breast cancer. Breast Cancer Res Treat.

[18] Santinelli A, Pisa E, Stramazzotti D, Fabris G (2008). HER-2 status discrepancy between primary breast cancer and metastatic sites. Impact on target therapy. Int J Cancer.

[19] Gonzalez-Angulo AM, Iwamoto T, Liu S (2012). Gene expression, molecular class changes, and pathway analysis after neoadjuvant systemic therapy for breast cancer. Clin Cancer Res.

[20] Aitken SJ, Thomas JS, Langdon SP (2010). Quantitative analysis of changes in ER, PR and HER2 expression in primary breast cancer and paired nodal metastases. Ann Oncol.

[21] Kroigard AB, Larsen MJ, Thomassen M, Kruse TA (2016). Molecular concordance between primary breast cancer and matched metastases. Breast J.

[22] Yao ZX, LJ L, Wang RJ (2014). Discordance and clinical significance of ER, PR, and HER2 status between primary breast cancer and synchronous axillary lymph node metastasis. Med Oncol.

[23] Onstenk W, Sieuwerts AM, Weekhout M (2015). Gene expression profiles of circulating tumor cells versus primary tumors in metastatic breast cancer. Cancer Lett.

[24] Aktas B, Kasimir-Bauer S, Muller V (2016). Comparison of the HER2, estrogen and progesterone receptor expression profile of primary tumor, metastases and circulating tumor cells in metastatic breast cancer patients. BMC Cancer.

[25] Vanharanta S, Massague J (2013). Origins of metastatic traits. Cancer Cell.

[26] Demeulemeester J, Kumar P, Moller EK (2016). Tracing the origin of disseminated tumor cells in breast cancer using single-cell sequencing. Genome Biol.

[27] Wolff AC, Hammond ME, Hicks DG (2013). Recommendations for human epidermal growth factor receptor 2 testing in breast cancer: American Society of Clinical Oncology/College of American Pathologists clinical practice guideline update. J Clin Oncol.

